# Evaluating the Efficacy and Safety of Anidulafungin and Caspofungin in Invasive Candida Infections in Intensive Care Units in India: A Prospective, Observational, Multicenter, Open-Label Study

**DOI:** 10.7759/cureus.88039

**Published:** 2025-07-15

**Authors:** Yatin Mehta, Polati Vishnu Rao, Rajesh Mahajan, Charles Panackel

**Affiliations:** 1 Critical Care and Anesthesiology, Medanta - The Medicity, Gurugram, IND; 2 Infectious Diseases, Apollo Hospitals, Hyderabad, IND; 3 Medicine, Dayanand Medical College and Hospital, Ludhiana, IND; 4 Integrated Liver Care, Aster Medcity, Kochi, IND

**Keywords:** anidulafungin, anti-fungal, caspofungin, critical care, invasive candida infection

## Abstract

Background: Invasive Candida infections are a significant concern in ICU patients, with mortality rates ranging from 30-50%. This study aimed to assess the demographic and risk factors, as well as the safety and efficacy of anidulafungin and caspofungin in treating invasive Candida infections in ICU patients.

Methodology: This prospective, observational, multicenter, open-label study was conducted across four hospitals in India to evaluate the safety and efficacy of anidulafungin and caspofungin in adult ICU patients with confirmed or suspected invasive Candida infections. Patients aged ≥18 years were treated with either anidulafungin or caspofungin. Data were collected on sociodemographic characteristics, medical history, medications, and clinical outcomes, including hospital stay, treatment duration, and clinical and microbiological responses.  Efficacy assessment is based on clinical and microbiologic success. Clinical success is defined as the resolution of signs and symptoms of invasive candidiasis, and microbiologic success is defined as the eradication of Candida species present at baseline, as determined on follow-up culture. A positive global response refers to both clinical and microbiologic success.

Results: A total of 44 patients received anidulafungin and 18 received caspofungin. The mean age for anidulafungin-treated patients was 61.1±14.1 years, while for caspofungin-treated patients, it was 56.6±14.3 years. Anidulafungin achieved a clinical success rate of 34.9%, microbiological success of 34.9%, and a global response rate of 46.5%. Caspofungin showed clinical success of 50%, microbiological success of 70.7%, and a global response rate of 47.1%.

Conclusions: The study showed that anidulafungin is effective in treating invasive candida infections and could be an equivalent alternative in terms of efficacy.

## Introduction

Candidiasis refers to cutaneous, mucosal, and deep-seated organ infections caused by fungi of the *Candida* genus. Invasive candidiasis is a bloodstream infection with *Candida* species that spreads to other organs [1]. The annual incidence rate of candidemia is 1,88,000 [2]. An epidemiological study in India reported an incidence of 6.51 cases of intensive care unit (ICU)-acquired candidemia per 1000 ICU admissions, with a median ICU stay of eight days. *Candida auris* (*C. auris*) is the most common isolate from Indian ICUs, followed by *C. tropicalis* and *C. parapsilosis*. *C. auris* was reported to have a high resistance to azoles and polyenes. It has a higher mortality compared to other *Candida* species. Echinocandin monotherapy is as effective as other antifungals, so routine combination therapy is not indicated in most cases [2]. Among the *Candida *species, *C. albicans*, *C. glabrata*, *C. tropicalis*, *C. parapsilosis*, and *C. krusei* cause more than 90% of invasive infections. The type of infection depends on the geographical location, patient population, and clinical settings. The National Nosocomial Infections Surveillance System (NNISS) reports *Candida* species as the fourth most common nosocomial bloodstream pathogen [3]. Estimated mortality rates are as high as 45%, probably due to delayed diagnosis and inappropriate initial antifungal therapies [3].

Compared with fluconazole, echinocandins are fungicidal with a broader spectrum of activity, a favorable safety profile, and fewer drug-drug interactions. The European Society of Clinical Microbiology and Infectious Diseases (ESCMID) and the Infectious Diseases Society of America (IDSA) guidelines recommend echinocandins for initial candidiasis therapy [4].

As the incidence of fungal infections caused by fluconazole-resistant non-C. albicans species is increasing, and echinocandins have become important first-line antifungals in patients infected with this species. Among the echinocandins, anidulafungin and caspofungin are approved for treating invasive candidiasis in adults [5].

Prior to the start of the study, a baseline evaluation was carried out to identify the echinocandins most prescribed at the study sites. Anidulafungin and caspofungin were found to be the most frequently used. Hence, the objective of the study was to evaluate the demographic characteristics, risk factors, and safety and efficacy of anidulafungin and caspofungin prescribed for invasive *Candida *infections in ICUs.

## Materials and methods

This was a prospective, observational, multicenter, open-label study to evaluate the safety and efficacy of anidulafungin and caspofungin in adult subjects with invasive *Candida* infection in the ICU of four hospitals across India. Institutional review board and ethics committee approval were obtained at each center: (1) Drugs Trial Ethics Committee (DTEC), Dayanand Medical College and Hospital (Ref. No.: DMCH/DTEC/2022/1280); (2) Aster Medcity, Institutional Ethics Committee (IEC) (Ref. No.: AM/EC/313(A)-2023); (3) IEC, Biomedical Research, Apollo Hospitals (Ref. No.: AHJ-C-S-002/04-21); and (4) Medanta IEC, Medanta - The Medicity (Ref. No.: 1355/2021). The study was conducted over two and a half months, from November 2023 to February 2024.

Before being enrolled in the study, subjects provided written consent authorizing the investigator to use or disclose personal and/or clinical data. Subjects of either gender, aged≥18 years, with a confirmed/ suspected case of invasive *Candida* infection, who were able to provide informed consent and were prescribed caspofungin or anidulafungin, were included in the study. Patients were excluded from the study if they were younger than 18 years, had infections caused by *Aspergillus*, other molds, or yeasts such as *Cryptococcus*, were being treated with micafungin or other antifungal medications, or were pregnant or breastfeeding. Data on sociodemographic characteristics (gender and age), physical examination (height, weight, body mass index (BMI), blood pressure, and heart rate), comorbidities, and concomitant medications were recorded. The patient's clinical response and biochemical parameters, including complete blood count, renal and liver functions, treatment duration, and hospital stay, were recorded. The appropriate specimens for microbiology confirmation were sent before initiating the treatment, and only subjects with positive culture reports continued in the study and were entered into the clinical research form (CRF).

This being a real-world observational study, no additional tests or interventions were suggested; however, routine investigations performed as a part of ICU clinical protocol and as per the investigator's discretion were captured in the CRF. The data was collected from all the enrolled subjects from four centers in India. Subjects received either anidulafungin or caspofungin treatment for invasive Candida infection as per the investigator's clinical judgment.

A global response was considered successful if there was both clinical and microbiologic success. Clinical success was defined as the resolution of signs and symptoms of invasive candidiasis and the absence of the need for additional systemic antifungal therapy. Microbiologic success was defined as the eradication of *Candida *species present at baseline, as determined by follow-up culture.

Statistical analysis

Descriptive analysis was performed on the collected data. Subject demographics, medical and surgical history, prior and concomitant medication, risk factors for invasive candidiasis, duration of treatment and number of hospital days, and clinical, microbiological, and global responses were summarized with frequency (n) and percentages (%) for patients receiving anidulafungin and caspofungin.

## Results

Anidulafungin

Patient Characteristics

The mean age of subjects on anidulafungin was 61.1±14.1 years. There were 31 (70.5%) male subjects and 13 (29.5%) female subjects. The mean hospital stay was 11.1±7.5 days, and the mean duration of treatment was 18.3±6.7 days (Table 1).

**Table 1:** Characteristics of patients on anidulafungin

Patient characteristics	Anidulafungin (N=44)
Age (Mean± SD)	61.1±14.1 years
Gender, n (%)	
Male	31 (70.5)
Female	13 (29.5)
BMI (n=20) (Mean± SD)	27.0±5.6 kg/m2
Number of hospital days	11.1±7.5
Duration of treatment	18.3±6.7

*Summary of Medical History, Medications, and Risk Factors* 

Among patients on anidulafungin, 81.8% of subjects had a medical or surgical history, with diabetes mellitus being the most common comorbidity (see Appendix A). Prior medications were used by 90.9% of subjects, with the majority taking antibiotics and proton pump inhibitors. Along with anidulafungin and caspofungin, most of the subjects were taking antibiotics, followed by diuretics as concomitant medication (see Appendix B). The most common risk factor was an indwelling central venous catheter (72.7%), followed by a urinary catheter (70.5%) and recent antibiotic use (61.4%) (Table 2).

**Table 2:** Summary of subjects with risk factors for invasive Candida infection (on anidulafungin)

Risk factors	Anidulafungin (N=44) n (%)
Central venous catheter	32 (72.7)
Immunosuppression	8 (18.2)
Inadequate therapy	2 (4.5)
Diabetes mellitus	9 (20.5)
Invasive mechanical ventilation	26 (59.1)
Multifocal Candida colonization	10 (22.7)
No risk factor	6 (13.6)
Recent antibiotic use	27 (61.4)
Renal replacement therapy	7 (15.9)
Severe sepsis or septic shock	19 (43.2)
Surgery	18 (40.9)
Total parenteral nutrition	23 (52.3)
Urinary catheter	31 (70.5)

Safety and Efficacy of Anidulafungin in Invasive Candidiasis

Global response was found to be 32.4% (11 of 34 subjects) at the second assessment, 60.0% (6 of 10 subjects) at the third assessment, and 34.9% (15 of 43 subjects) at the end of treatment. Clinical success was 33% (11 of 33 subjects) at the second assessment, 66.7% (6 of 9 subjects) at the third assessment, and 34.9% (15 of 43 subjects) at the end of treatment. Microbiologic success was 38.2% (13 of 34 subjects) at the second assessment, 60% (6 of 10 subjects) at the third assessment, and 46.5% (20 of 43 subjects) at the end of treatment (Table 3).

**Table 3:** Clinical efficacy and safety of anidulafungin

Echinocandin	End of treatment response		
-	Clinical success	Microbiological success	Global response
Anidulafungin	34.9%	34.9%	46.5%

Two serious adverse events (AEs) were observed in one subject with no reasonable possibility of a relationship between these AEs and anidulafungin.

Caspofungin

Summary of Demographics 

The mean age of subjects on caspofungin was 56.6±14.3 years. There were more male subjects than female subjects. The mean hospital stay was 10.1±7.5 days, and the mean duration of treatment was 11.8±7.2 days (Table 4).

**Table 4:** Characteristics of patients on caspofungin

Patient characteristics	Caspofungin (N=18)
Age (Mean± SD)	56.6±14.3 years
Gender, n (%)	
Male	10 (55.6)
Female	8 (44.4)
BMI (n=14) (Mean± SD)	25.1±4.2 kg/m2
Number of hospital days	10.1±7.5
Duration of treatment	11.8±7.2

*Summary of Medical History, Medications, and Risk Factors* 

Among patients on caspofungin, 72.2% of subjects had a medical or surgical history, with type 2 diabetes mellitus being the most common. Prior medications were used by 77.8% of subjects, with the majority taking antibiotics and proton pump inhibitors. Along with caspofungin, most of the subjects were taking antibiotics, followed by diuretics as concomitant medication (see Appendix B). The most common risk factors were recent antibiotic use (77.8%) and urinary catheter (77.8%), followed by total parenteral nutrition (66.7%) and severe sepsis or septic shock (61.1%) (Table 5).

**Table 5:** Summary of subjects with risk factors for invasive Candida infection (on caspofungin)

Risk factors	Caspofungin (N=18) n (%)
Central venous catheter	10 (55.6)
Immunosuppression	4 (22.2)
Diabetes mellitus	3 (16.7)
Invasive mechanical ventilation	7 (38.9)
Multifocal Candida colonization	2 (11.1)
No risk factor	0
Recent antibiotic use	14 (77.8)
Renal replacement therapy	2 (11.1)
Severe sepsis or septic shock	11 (61.1)
Surgery	6 (33.3)
Total parenteral nutrition	12 (66.7)
Urinary catheter	14 (77.8)

Safety and Efficacy of Caspofungin in Invasive Candidiasis

Global response was found to be 50% (7 of 14 subjects) at second assessment, 33.3% (1 of 3 subjects) at third assessment, and 47.1% (8 of 17 subjects) at the end of treatment. Clinical success was 50% (7 of 14 subjects) at the second assessment, 33% (1 of 3 subjects) at the third assessment, and 50% (8 of 16 subjects) at the end of treatment. Microbiologic success was 78.6% (11 of 14 subjects) at second assessment, 33.3% (1 of 3 subjects) at third assessment, and 70.6% (12 of 17 subjects) at the end of treatment (Table 6).

**Table 6:** Clinical efficacy and safety of caspofungin

Echinocandin	End of treatment response		
-	Clinical success	Microbiological success	Global response
Caspofungin	50%	70.7%	47.1%

There were no AEs reported in subjects from the caspofungin group.

## Discussion

Over 1.5 million people die due to fungal infections worldwide [5]. A literature search from 2010 to 2023 showed an annual incidence of 6.5 million invasive fungal infections and 3.8 million deaths [6]. Invasive candidiasis, which is the most common hospital-acquired infection, is linked to high mortality rates, often due to delays in recognition and the start of suitable antifungal treatment. The present study assessed the efficacy and safety of treatment prescribed for ICU subjects with invasive *Candida *infections.

Treatment approaches for invasive candidiasis include prophylaxis, which is risk-driven, or pre-emptive based on colonization or biomarker-driven, empirical, which is fever-driven, and targeted therapy, which is culture-driven. There are several guidelines; however, based on the host factors, distribution of local species, and antifungal resistance, the clinicians tailor and prescribe the therapy [7]. Echinocandins are recommended as first-line agents for invasive candidiasis because they have broad-spectrum activity with a good safety profile and are clinically superior to other systemic antifungals [8]. Because they affect fungal cell walls, which are different from human cells, echinocandins are a special class of antifungals with the lowest systemic toxicity. The three drugs in this group, micafungin, caspofungin, and anidulafungin, are equally effective [9]. This study aimed to assess how well each treatment reduced infection, as well as to evaluate the side effect profiles of each treatment.

It was observed that anidulafungin was preferred and prescribed more frequently compared to caspofungin for invasive *Candida *infection, leading to a smaller sample size of the caspofungin group. A retrospective analysis of the efficacy of echinocandin on a subgroup of ICU patients showed favorable results for anidulafungin. It was observed that anidulafungin can benefit critically ill patients clinically and economically [10].

Our study showed that the most common risk factors for invasive *Candida *infection were a central venous catheter and prior use of antibiotics (see Appendix C). This is in line with other studies in the literature where common risk factors for candida infections were central venous catheter, use of broad-spectrum antibiotics, parenteral nutrition, and immunosuppression (such as cancer chemotherapy) [8,11].

The recommended duration of systemic antifungal therapy for candidemia (the most common form of invasive candidiasis) is at least 14 days after the eradication of *Candida *spp. from the blood and the resolution of all symptoms and signs of infection. At the same time, it may vary in deep-seated invasive *Candida* infections from several weeks to 6-12 months [12]. In this study, the median duration of treatment was 23 days and 10 days with anidulafungin and caspofungin, respectively.

Both anidulafungin and caspofungin were well tolerated in our study, and there was no treatment discontinuation due to toxicity. The side effects of treatment with echinocandins are comparable to side effects observed using fluconazole, and less significant than amphotericin B. Gastrointestinal problems, such as nausea, vomiting, and diarrhea, occur in less than 7% of subjects. The incidence of phlebitis is 3-25% with caspofungin and less than 2% with anidulafungin [13]. Anemia, leukopenia, neutropenia, and thrombocytopenia account for less than 10% of all side effects. Side effects leading to discontinuation of the drug occur less frequently than with other antifungal drugs. In the present study, biochemical data were available only for a few subjects, so it is not possible to generalize this data and conclude on the toxicity of echinocandins. However, there was a transient increase in liver enzymes in both anidulafungin- and caspofungin-treated patients at baseline and following assessments, as reported in the literature [14].

A total of two AEs were observed in one subject from the anidulafungin group, while no AEs were reported in subjects from the caspofungin group. This difference in AEs may be attributed to anidulafungin being prescribed more frequently than caspofungin, which also contributes to the smaller sample size of the caspofungin group.

The limitations of the study were that, being a Real-World Study (RWS), laboratory assessments and other data could not be captured for all the subjects, and the treatment prescribed was based on physician’s choice, whereby a more significant number of patients were prescribed anidulafungin leading to potential observer or treatment bias especially in subjective outcome assessments. Due to a sample size difference and lack of randomization, a comparison between the treatment groups was not possible.  Potential selection bias and lack of adequate follow-up are factors that may influence the outcome of the study.

## Conclusions

The study evaluated the safety and efficacy of anidulafungin and caspofungin in invasive *Candida* infections in ICU patients. Both were found to be effective in treating invasive *Candida* infections. There were no discontinuations, which shows that both these echinocandins were well tolerated. The central venous catheters and prior antibiotic use were the major risk factors. Higher prescription rate of anidulafungin causes an imbalance in the sample size. Further controlled trials might provide more data on the safety and efficacy of anidulafungin and caspofungin.

## Appendices

Appendix A 

**Table 7:** Summary medical and surgical history Percentages were computed using N provided in the column header. SOC: system organ class; n: number of subjects; E: number of medical history events

System organ class/preferred term	Anidulafungin (N=44)		Caspofungin (N=18)		Overall (N=6)	
	n (%)	E	n (%)	E	n (%)	E
All SOCs	36 (81.8)	92	13 (72.2)	33	49 (79.0)	125
Cardiac disorders	3 (6.8)	3	1 (5.6)	1	4 (6.5)	4
-Atrial fibrillation	1 (2.3)	1	0	0	1 (1.6)	1
-Coronary artery disease	2 (4.5)	2	0	0	2 (3.2)	2
-Left ventricular dysfunction	0	0	1 (5.6)	1	1 (1.6)	1
Endocrine disorders	4 (9.1)	4	1 (5.6)	1	5 (8.1)	5
-Hypothyroidism	3 (6.8)	3	1 (5.6)	1	4 (6.5)	4
-Thyroid disorder	1 (2.3)	1	0	0	1 (1.6)	1
Eye disorders	1 (2.3)	1	0	0	1 (1.6)	1
-Diabetic retinopathy	1 (2.3)	1	0	0	1 (1.6)	1
Gastrointestinal disorders	2 (4.5)	2	1 (5.6)	2	3 (4.8)	4
-Gastric ulcer	0	0	1 (5.6)	1	1 (1.6)	1
-Gastrooesophageal reflux disease	1 (2.3)	1	0	0	1 (1.6)	1
-Pancreatolithiasis	1 (2.3)	1	0	0	1 (1.6)	1
-Upper gastrointestinal haemorrhage	0	0	1 (5.6)	1	1 (1.6)	1
Hepatobiliary disorders	5 (11.4)	10	2 (11.1)	2	7 (11.3)	12
-Acute on chronic liver failure	2 (4.5)	2	0	0	2 (3.2)	2
-Bile duct stone	1 (2.3)	1	0	0	1 (1.6)	1
-Cholelithiasis	1 (2.3)	1	0	0	1 (1.6)	1
-Hepatic cirrhosis	2 (4.5)	2	2 (11.1)	2	4 (6.5)	4
-Hepatorenal syndrome	1 (2.3)	1	0	0	1 (1.6)	1
-Liver disorder	1 (2.3)	1	0	0	1 (1.6)	1
-Portal hypertension	1 (2.3)	1	0	0	1 (1.6)	1
-Portal vein thrombosis	1 (2.3)	1	0	0	1 (1.6)	1
Immune system disorders	1 (2.3)	1	0	0	1 (1.6)	1
-Hemophagocytic lymphohistiocytosis	1 (2.3)	1	0	0	1 (1.6)	1
Infections and infestations	8 (18.2)	11	3 (16.7)	5	11 (17.7)	16
-Candida infection	0	0	1 (5.6)	1	1 (1.6)	1
-Hepatitis A	1 (2.3)	1	0	0	1 (1.6)	1
-Hepatitis B	1 (2.3)	1	0	0	1 (1.6)	1
-Kidney infection	1 (2.3)	1	0	0	1 (1.6)	1
-Peritonitis bacterial	1 (2.3)	1	0	0	1 (1.6)	1
-Pneumonia	1 (2.3)	1	0	0	1 (1.6)	1
-Poliomyelitis	0	0	1 (5.6)	1	1 (1.6)	1
-Pulmonary tuberculosis	1 (2.3)	1	0	0	1 (1.6)	1
-Sepsis	1 (2.3)	1	1 (5.6)	1	2 (3.2)	2
-Systemic candida	1 (2.3)	1	0	0	1 (1.6)	1
-Tuberculosis	1 (2.3)	1	0	0	1 (1.6)	1
-Urinary tract infection	2 (4.5)	2	1 (5.6)	1	3 (4.8)	3
-Varicella	0	0	1 (5.6)	1	1 (1.6)	1
Investigations	1 (2.3)1	1	2 (11.1)	2	3 (4.8)	3
-Angiocardiogram	0	0	2 (11.1)	2	2 (3.2)	2
-Positron emission tomogram	1 (2.3)	1	0	0	1 (1.6)	1
Metabolism and nutrition disorders	16 (36.4)	17	4 (22.2)	4	20 (32.3)	21
-Dyslipidaemia	1 (2.3)	1	0	0	1 (1.6)	1
-Hypokalaemia	1 (2.3)	1	0	0	1 (1.6)	1
-Type 1 diabetes mellitus	4 (9.1)	4	1 (5.6)	1	5 (8.1)	5
-Type 2 diabetes mellitus	11 (25.0)	11	3 (16.7)	3	14 (22.6)	14
Musculoskeletal and connective tissue disorders	2 (4.5)	2	0	0	2 (3.2)	2
-Scleroderma	1 (2.3)	1	0	0	1 (1.6)	1
-Systemic lupus erythematosus	1 (2.3)	1	0	0	1 (1.6)	1
Neoplasms benign, malignant and unspecified (including cysts and polyps)	5 (11.4)	5	2 (11.1)	2	7 (11.3)	7
-Anal cancer	1 (2.3)	1	0	0	1 (1.6)	1
-Invasive ductal breast carcinoma	1 (2.3)	1	0	0	1 (1.6)	1
-Lung neoplasm malignant	1 (2.3)	1	0	0	1 (1.6)	1
-Ovarian cancer	0	0	1 (5.6)	1	1 (1.6)	1
-Prostate cancer	1 (2.3)	1	1 (5.6)	1	2 (3.2)	2
-Transitional cell carcinoma	1 (2.3)	1	0	0	1 (1.6)	1
Nervous system disorders	2 (4.5)	2	0	0	2 (3.2)	2
-Hepatic encephalopathy	1 (2.3)	1	0	0	1 (1.6)	1
-Metabolic encephalopathy	1 (2.3)	1	0	0	1 (1.6)	1
Renal and urinary disorders	5 (11.4)	5	0	0	5 (8.1)	5
-Acute kidney injury	1 (2.3)	1	0	0	1 (1.6)	1
-Chronic kidney disease	3 (6.8)	3	0	0	3 (4.8)	3
-Nephropathy	1 (2.3)	1	0	0	1 (1.6)	1
Reproductive system and breast disorders	0	0	1 (5.6)	1	1 (1.6)	1
-Benign prostatic hyperplasia	0	0	1 (5.6)	1	1 (1.6)	1
Respiratory, thoracic and mediastinal disorders	0	0	3 (16.7)	3	3 (4.8)	3
-Chronic obstructive pulmonary disease	0	0	2 (11.1)	2	2 (3.2)	2
-Chronic respiratory disease	0	0	1 (5.6)	1	1 (1.6)	1
Skin and subcutaneous tissue disorders	0	0	1 (5.6) 1	1 (5.6) 1	1 (1.6)	1
-Psoriasis	0	0	1 (5.6)1	1 (5.6) 1	1 (1.6)	1
Surgical and medical procedures	11 (25.0)	13	7 (38.9) 8	7 (38.9) 8	18 (29.0)	21
-Abdominal cavity drainage	1 (2.3)	1	1 (5.6) 1	1 (5.6) 1	2 (3.2)	2
-Coronary angioplasty	1 (2.3)	1	1 (5.6) 1	1 (5.6) 1	2 (3.2)	2
-Coronary artery bypass	2 (4.5)	2	1 (5.6) 1	1 (5.6) 1	3 (4.8)	3
-Debridement	1 (2.3)	1	0	0	1 (1.6)	1
-Decompressive craniectomy	0	0	1 (5.6) 1	1 (5.6) 1	1 (1.6)	1
-Explorative laparotomy	0	0	2 (11.1) 2	2 (11.1) 2	2 (3.2)	2

Appendix B 

**Table 8:** Summary of prior medication

Pharmacological class/standard name	Anidulafungin (N=44)		Caspofungin (N=18)		Overall (N=62)	
	n (%)	E	n (%)	E	n (%)	E
All pharmacological class	40 (90.9)	228	14 (77.8)	96	54 (87.1)	324
Alpha blocker	0	0	1 (5.6)	1	1 (1.6)	1
-Tamsulosin	0	0	1 (5.6)	1	1 (1.6)	1
Alpha-adrenergic agonist	1 (2.3)	1	0	0	1 (1.6)	1
-Midodrine	1 (2.3)	1	0	0	1 (1.6)	1
Alpha-blocker + 5-alpha-reductase inhibitor	1 (2.3)	1	0	0	1 (1.6)	1
-Silodosin + dutasteride	1 (2.3)	1	0	0	1 (1.6)	1
Amino acid	2 (4.5)	2	1 (5.6)	1	3 (4.8)	3
-Ademetionine	1 (2.3)	1	0	0	1 (1.6)	1
-Albumin	1 (2.3)	1	1 (5.6)	1	2 (3.2)	2
Amino acid + amino acid	2 (4.5)	2	0	0	2 (3.2)	2
-L-ornithine + L-aspartate	2 (4.5)	2	0	0	2 (3.2)	2
Amino acid + amino acid + digestive enzyme	0	0	1 (5.6)	1	1 (1.6)	1
-L-ornithine + L-aspartate + pancreatin	0	0	1 (5.6)	1	1 (1.6)	1
Amino acid + vitamin B5 + vitamin B12 + vitamin B6	2 (4.5)	2	3 (16.7)	3	5 (8.1)	5
-L-lysine + D-panthenol + vitamin B12 + vitamin B6	2 (4.5)	2	3 (16.7)	3	5 (8.1)	5
Analgesic/antipyretic	0	0	1 (5.6)	1	1 (1.6)	1
-Paracetamol	0	0	1 (5.6)	1	1 (1.6)	1
Angiotensin receptor blocker + calcium channel blocker + diuretic	0	0	1 (5.6)	1	1 (1.6)	1
-Telmisartan + amlodipine + hydrochlorothiazide	0	0	1 (5.6)	1	1 (1.6)	1
Angiotensin receptor blocker	1 (2.3)	1	0	0	1 (1.6)	1
-Telmisartan	1 (2.3)	1	0	0	1 (1.6)	1
Anti-anginal	1 (2.3)	1	0	0	1 (1.6)	1
-Trimetazidine	1 (2.3)	1	0	0	1 (1.6)	1
Anti-arrhythmic	3 (6.8)	3	0	0	3 (4.8)	3
-Amiodarone	3 (6.8)	3	0	0	3 (4.8)	3
Anti-spasmodic	1 (2.3)	1	0	0	1 (1.6)	1
-Flavoxate	1 (2.3)	1	0	0	1 (1.6)	1
Anti-ulcer	0	0	1 (5.6)	1	1 (1.6)	1
-Sucralfate	0	0	1 (5.6)	1	1 (1.6)	1
Antibiotic	29 (65.9)	62	11 (61.1)	23	40 (64.5)	85
-Ampicillin	1 (2.3)	1	0	0	1 (1.6)	1
-Azithromycin	1 (2.3)	1	0	0	1 (1.6)	1
-Aztreonam	6 (13.6)	6	0	0	6 (9.7)	6
-Biapenem	1 (2.3)	1	1 (5.6)	1	2 (3.2)	2
-Cefixime	1 (2.3)	1	0	0	1 (1.6)	1
-Ceftazidime	1 (2.3)	1	0	0	1 (1.6)	1
-Ceftriaxone	1 (2.3)	1	0	0	1 (1.6)	1
-Clindamycin	2 (4.5)	2	1 (5.6)	1	3 (4.8)	3
-Colistimethate sodium	3 (6.8)	3	0	0	3 (4.8)	3
-Daptomycin	1 (2.3)	1	0	0	1 (1.6)	1
-Doripenem	1 (2.3)	1	0	0	1 (1.6)	1
-Doxycycline	1 (2.3)	1	0	0	1 (1.6)	1
-Flucloxacillin sodium	1 (2.3)	1	0	0	1 (1.6)	1
-Fosfomycin	2 (4.5)	2	0	0	2 (3.2)	2
-Imipenem	1 (2.3)	1	2 (11.1)	2	3 (4.8)	3
-Levofloxacin	1 (2.3)	1	0	0	1 (1.6)	1
-Levonadifloxacin	3 (6.8)	3	1 (5.6)	1	4 (6.5)	4
-Meropenem	11 (25.0)	11	6 (33.3)	6	17 (27.4)	17
-Meropenem + sulbactam	1 (2.3)	1	0	0	1 (1.6)	1
-Metronidazole	3 (6.8)	3	2 (11.1)	2	5 (8.1)	5
-Minocycline	3 (6.8)	3	0	0	3 (4.8)	3
-Polymyxin B	7 (15.9)	7	0	0	7 (11.3)	7
-Rifaximin	2 (4.5)	2	3 (16.7)	3	5 (8.1)	5
-Teicoplanin	7 (15.9)	7	6 (33.3)	1	13 (21.0)	13
-Tigecycline	0	0	1 (5.6)	1	1 (1.6)	1
Antibiotic + beta-lactamase inhibitor	4 (9.1)	4	1 (5.6)	1	5 (8.1)	5
-Cefpirome + sulbactam	1 (2.3)	1	0	0	1 (1.6)	1
-Piperacillin + tazobactum	3 (6.8)	3	1 (5.6)	1	4 (6.5)	4
Antibiotic + adjuvant + beta-lactamase inhibitor	6 (13.6)	6	1 (5.6)	1	7 (11.3)	7
-Ceftriaxone + disodium edetate + sulbactam	6 (13.6)	6	1 (5.6)	1	7 (11.3)	7
Antibiotic + non-beta-lactam beta-lactamase inhibitor	9 (20.5)	9	0	0	9 (14.5)	9
-Ceftazidime + avibactam	9 (20.5)	9	0	0	9 (14.5)	9
Anticoagulant	8 (18.2)	9	3 (16.7)	4	11 (17.7)	13
-Apixaban	0	0	1 (5.6)	1	1 (1.6)	1
-Dalteparin	7 (15.9)	7	0	0	7 (11.3)	7
-Enoxaparin	2 (4.5)	2	3 (16.7)	3	5 (8.1)	5
Antidiabetic	0	0	1 (5.6)	3	1 (1.6)	3
-Gliclazide	0	0	1 (5.6)	1	1 (1.6)	1
-Metformin	0	0	1 (5.6)	1	1 (1.6)	1
-Vildagliptin	0	0	1 (5.6)	1	1 (1.6)	1
Antiemetic	5 (11.4)	5	1 (5.6)	1	6 (9.7)	6
-Ondansetron	5 (11.4)	5	1 (5.6)	1	6 (9.7)	6
Antiepileptic	8 (18.2)	14	4 (22.2)	5	12 (19.4)	19
-Brivaracetam	0	0	1 (5.6)	1	1 (1.6)	1
-Clobazam	1 (2.3)	1	0	0	1 (1.6)	1
-Clonazepam	1 (2.3)	1	1 (5.6)	1	2 (3.2)	2
-Gabapentin	1 (2.3)	1	0	0	1 (1.6)	1
-Lacosamide	1 (2.3)	1	0	0	1 (1.6)	1
-Levetiracetam	6 (13.6)	6	2 (11.1)	2	8 (12.9)	8
-Perampanel	2 (4.5)	2	0	0	2 (3.2)	2
-Pregabalin	0	2	1 (5.6)	1	1 (1.6)	1
-Sodium valproate	2 (4.5)	2	0	0	2 (3.2)	2
Antifungal	2 (4.5)	2	0	0	2 (3.2)	2
-Clotrimazole	1 (2.3)	1	0	0	1 (1.6)	1
-Fluconazole	1 (2.3)	1	0	0	1 (1.6)	1
Antineoplastic	0	0	1 (5.6)	1	1 (1.6)	1
-Abiraterone acetate	0	0	1 (5.6)	1	1 (1.6)	1
Antioxidant	2 (4.5)	2	1 (5.6)	1	3 (4.8)	3
-Glutathione	2 (4.5)	2	1 (5.6)	1	3 (4.8)	3
Antiplatelet	0	0	1 (5.6)	1	1 (1.6)	1
-Clopidogrel	0	0	1 (5.6)	1	1 (1.6)	1
Antirheumatic	1 (2.3)	1	0	0	1 (1.6)	1
-Hydroxychloroquine	1 (2.3)	1	0	0	1 (1.6)	1
Antitubercular	1 (2.3)	4	0	0	1 (1.6)	4
-Ethambutol	1 (2.3)	1	0	0	1 (1.6)	1
-Isoniazid	1 (2.3)	1	0	0	1 (1.6)	1
-Pyrazinamide	1 (2.3)	1	0	0	1 (1.6)	1
-Rifampin + isoniazid + pyrazinamide + ethambutol	1 (2.3)	1	0	0	1 (1.6)	1
Antiviral	0	0	1 (5.6)	1	1 (1.6)	1
-Acyclovir	0	0	1 (5.6)	1	1 (1.6)	1
Atypical antipsychotic	3 (6.8)	3	0	0	3 (4.8)	3
-Aripiprazole	1 (2.3)	1	0	0	1 (1.6)	1
-Levosulpiride	1 (2.3)	1	0	0	1 (1.6)	1
-Quetiapine	1 (2.3)	1	0	0	1 (1.6)	1
Beta 3-receptor agonist	1 (2.3)	1	0	0	1 (1.6)	1
-Mirabegron	1 (2.3)	1	0	0	1 (1.6)	1
Beta 3-receptor agonist + antimuscarinic	0	0	1 (5.6)	1	1 (1.6)	1
-Mirabegron + solifenacin	0	0	1 (5.6)	1	1 (1.6)	1
Beta blocker	2 (4.5)	2	1 (5.6)	1	3 (4.8)	3
-Bisoprolol	2 (4.5)	2	1 (5.6)	1	3 (4.8)	3
Beta-2 adrenergic receptor agonist + mucolytic/bronchodilator + expectorant	1 (2.3)	1	1 (5.6)	1	2 (3.2)	2
-Terbutaline + acebrophylline + guaifenesin	1 (2.3)	1	1 (5.6)	1	2 (3.2)	2
Bronchodilator	2 (4.5)	2	1 (5.6)	1	3 (4.8)	3
-Etofylline + theophylline	1 (2.3)	1	1 (5.6)	1	2 (3.2)	2
-Levosalbutamol	1 (2.3)	1	0	0	1 (1.6)	1
Bronchodilator + Anticholinergic	3 (6.8)	3	1 (5.6)	1	4 (6.5)	4
-Levosalbutamol + Ipratropium	3 (6.8)	3	1 (5.6)	1	4 (6.5)	4
Calcium channel blocker	4 (9.1)	4	2 (11.1)	2	6 (9.7)	6
-Amlodipine	3 (6.8)	3	1 (5.6)	1	4 (6.5)	4
-Diltiazem	1 (2.3)	1	1 (5.6)	1	2 (3.2)	2
Carbonic anhydrase inhibitor	0	0	1 (5.6)	1	1 (1.6)	1
-Acetazolamide	0	0	1 (5.6)	1	1 (1.6)	1
Corticosteroid	8 (18.2)	8	3 (16.7)	3	11 (17.7)	11
-Budesonide	1 (2.3)	1	2 (11.1)	2	3 (4.8)	3
-Hydrocortisone	7 (15.9)	7	1 (5.6)	1	8 (12.9)	8
Diuretic	7 (15.9)	7	1 (5.6)	1	8 (12.9)	8
-Furosemide	4 (9.1)	4	1 (5.6)	1	5 (8.1)	5
-Torasemide	3 (6.8)	3	0	0	3 (4.8)	3
Dopamine antagonist	0	0	1 (5.6)	1	1 (1.6)	1
-Domperidone	0	0	1 (5.6)	1	1 (1.6)	1
Dopamine precursor + peripheral decarboxylase inhibitor	2 (4.5)	2	0	0	2 (3.2)	2
-Levodopa + carbidopa	2 (4.5)	2	0	0	2 (3.2)	2
Essential mineral	2 (4.5)	2	1 (5.6)	1	3 (4.8)	3
-Magnesium oxide	1 (2.3)	1	0	0	1 (1.6)	1
-Potassium chloride	1 (2.3)	1	1 (5.6)	1	2 (3.2)	2
Essential mineral/alkalizer	0	0	1 (5.6)	1	1 (1.6)	1
-Sodium bicarbonate	0	0	1 (5.6)	1	1 (1.6)	1
Essential mineral	2 (4.5)	2	0	0	2 (3.2)	2
-Chromium chloride + copper sulphate + manganese sulphate + selenium + zinc sulphate	1 (2.3)	1	0	0	1 (1.6)	1
-Sodium + potassium + magnesium + chloride + acetate + gluconate	1 (2.3)	1	0	0	1 (1.6)	1
Glucocorticoid	1 (2.3)	1	0	0	1 (1.6)	1
-Methylprednisolone	1 (2.3)	1	0	0	1 (1.6)	1
HMG-CoA reductase inhibitor + non-steroidal anti-inflammatory drug/anti-platelet	1 (2.3)	1	1 (5.6)	1	2 (3.2)	2
-Atorvastatin + aspirin	1 (2.3)	1	1 (5.6)	1	2 (3.2)	2
HMG-CoA-reductase	2 (4.5)	2	1 (5.6)	1	3 (4.8)	3
-Atorvastatin	2 (4.5)	2	1 (5.6)	1	3 (4.8)	3
Hepatoprotective	3 (6.8)	3	1 (5.6)	1	4 (6.5)	4
-Ursodeoxycholic acid	3 (6.8)	3	1 (5.6)	1	4 (6.5)	4
Hyperpolarization-activated cyclic nucleotide-gated (HCN) channel blockers	2 (4.5)	2	0	0	2 (3.2)	2
-Ivabradine	2 (4.5)	2	0	0	2 (3.2)	2
Immunosuppressant	4 (9.1)	4	0	0	4 (6.5)	4
-Mycophenolate mofetil	1 (2.3)	1	0	0	1 (1.6)	1
-Tacrolimus	3 (6.8)	3	0	0	3 (4.8)	3
Laxative	4 (9.1)	4	5 (27.8)	5	9 (14.5)	9
-Isabgol	0	0	1 (5.6)	1	1 (1.6)	1
-Lactulose	3 (6.8)	3	2 (11.1)	2	5 (8.1)	5
-Sodium picosulfate + liquid paraffin + milk of magnesia	1 (2.3)	1	2 (11.1)	2	3 (4.8)	3
Leukotriene receptor antagonist	1 (2.3)	1	1 (5.6)	1	2 (3.2)	2
-Montelukast	1 (2.3)	1	1 (5.6)	1	2 (3.2)	2
Mucolytic	1 (2.3)	1	1 (5.6)	1	2 (3.2)	2
-Acetylcysteine	1 (2.3)	1	1 (5.6)	1	2 (3.2)	2
Mucolytic/bronchodilator	0	0	1 (5.6)	1	1 (1.6)	1
-Acebrophylline	0	0	1 (5.6)	1	1 (1.6)	1
N-methyl-D-aspartate receptor antagonist	1 (2.3)	1	0	0	1 (1.6)	1
-Memantine	1 (2.3)	1	0	0	1 (1.6)	1
Neuraminidase inhibitor	0	00	1 (5.6)	1	1 (1.6)	1
-Oseltamivir phosphate	0	0	1 (5.6)	1	1 (1.6)	1
Non-amphetamine central nervous system stimulant	0	0	1 (5.6)	1	1 (1.6)	1
-Modafinil	0	0	1 (5.6)	1	1 (1.6)	1
Non-steroidal anti-inflammatory drug/anti-platelet	4 (9.1)	4	1 (5.6)	1	5 (8.1)	5
-Aspirin	4 (9.1)	4	1 (5.6)	1	5 (8.1)	5
Opioid analgesic	1 (2.3)	1	1 (5.6)	1	2 (3.2)	2
-Tramadol	1 (2.3)	1	1 (5.6)	1	2 (3.2)	2
Phosphate binder	2 (4.5)	2	0	0	2 (3.2)	2
-Sevelamer	2 (4.5)	2	0	0	2 (3.2)	2
Probiotic	1 (2.3)	1	0	0	1 (1.6)	1 (1.6) 1
-Live freeze-dried lactic acid bacteria + bifidobacteria	1 (2.3)	1	0	0	1 (1.6)	1 (1.6) 1
Probiotic + vitamin B1 + vitamin B3 + vitamin B5 + vitamin B6 + vitamin B7 + vitamin B9 + vitamin B12 + essential mineral + essential amino acid	0	0	1 (5.6)	1	1 (1.6)	1 (1.6) 1
-Lactic acid bacillus + vitamin B1 + vitamin B3 + vitamin B5 + vitamin B6 + vitamin B7 + vitamin B9 + vitamin B12 + magnesium oxide + DL-methionine	0	0	1 (5.6)	1	1 (1.6)	1
Prokinetic dopamine antagonist	1 (2.3)	1	0	0	1 (1.6)	1
-Metoclopramide	1 (2.3)	1	0	0	1 (1.6)	1
Protease inhibitor	2 (4.5)	2	0	0	2 (3.2)	2
-Ulinastatin	2 (4.5)	2	0	0	2 (3.2)	2
Proton pump inhibitor	22 (50.0)	23	10 (55.6)	10	32 (51.6)	33
-Esomeprazole	1 (2.3)	1	2 (11.1)	2	3 (4.8)	3
-Pantoprazole	22 (50.0)	22	8 (44.4)	8	30 (48.4)	30
Thyroid hormone	3 (6.8)	3	1 (5.6)	1	4 (6.5)	4
-Thyroxine	3 (6.8)	3	1 (5.6)	1	4 (6.5)	4
Urine alkalizer	0	0	1 (5.6)	1	1 (1.6)	1
-Disodium hydrogen citrate	0	0	1 (5.6)	1	1 (1.6)	1
Vasopressin receptor agonist	0	0	1 (5.6)	1	1 (1.6)	1
-Terlipressin	0	0	1 (5.6)	1	1 (1.6)	1
Vitamin B1 + vitamin B3 + vitamin B5 + vitamin C + vitamin B2 + vitamin B6 + vitamin B12 + vitamin B7	0	0	1 (5.6)	1	1 (1.6)	1
-Thiamine monotrate + nicotinamide + calcium pantothenate + ascorbic acid + riboflavin + pyridoxine hydrochloride + cyanocobalamin + biotin	0	0	1 (5.6)	1	1 (1.6)	1
Vitamin B6 + vitamin B3 + vitamin B12 + vitamin B9 + essential mineral + essential mineral + essential mineral	1 (2.3)	1	0	0	1 (1.6)	1
-Pyridoxine hydrochloride + nicotinamide + cyanocobalamin + folic acid + chromium picolinate + selenious acid + zinc sulphate monohydrate	1 (2.3)	1	0	0	1 (1.6)	1
Vitamin B9 + vitamin B6 + vitamin B12 + essential mineral	1 (2.3)	1	0	0	1 (1.6)	1
-L-5 methyltetrahydrofolate + pyridoxal 5 phosphate + mecobalamin + calcium	1 (2.3)	1	0	0	1 (1.6)	1

Appendix C 

**Table 9:** Comparative summary of subjects with risk factors for invasive Candida infection in anidulafungin and caspofungin groups

Risk factors	Anidulafungin (N=44) n (%)	Caspofungin (N=18) n (%)
Central venous catheter	32 (72.7)	10 (55.6)
Immunosuppression	8 (18.2)	4 (22.2)
Inadequate therapy	2 (4.5)	0
Diabetes mellitus	9 (20.5)	3 (16.7)
Invasive mechanical ventilation	26 (59.1)	7 (38.9)
Multifocal Candida colonization	10 (22.7)	2 (11.1)
No risk factor	6 (13.6)	0
Recent antibiotic use	27 (61.4)	14 (77.8)
Renal replacement therapy	7 (15.9)	2 (11.1)
Severe sepsis or septic shock	19 (43.2)	11 (61.1)
Surgery	18 (40.9)	6 (33.3)
Total parenteral nutrition	23 (52.3)	12 (66.7)
Urinary catheter	31 (70.5)	14 (77.8)
